# Is use of opioid agonist treatment associated with broader primary healthcare use among men with recent injecting drug use histories following release from prison? A prospective cohort study

**DOI:** 10.1186/s12954-023-00773-2

**Published:** 2023-03-28

**Authors:** Michael Curtis, Anna L. Wilkinson, Paul Dietze, Ashleigh C. Stewart, Stuart A. Kinner, Rebecca J. Winter, Campbell Aitken, Shelley J. Walker, Reece D. Cossar, Tony Butler, Mark Stoové

**Affiliations:** 1 Disease Elimination Program, Public Health Discipline, Burnet Institute, 85 Commercial Road, Melbourne, VIC 3004 Australia; 2 School of Public Health and Preventive Medicine, Monash University, Melbourne, VIC Australia; 3 Monash Addition Research Centre, Monash University, Melbourne, VIC Australia; 4 National Drug Research Institute, Curtin University, Melbourne, Australia; 5 School of Population Health, Curtin University, Perth, WA Australia; 6 Justice Health Unit, Melbourne School of Population and Global Health, University of Melbourne, Melbourne, VIC Australia; 7 Centre for Adolescent Health, Murdoch Children’s Research Institute, Melbourne, VIC Australia; 8 Griffith Criminology Institute, Griffith University, Brisbane, QLD Australia; 9 Department of Gastroenterology, St Vincent’s Hospital, Melbourne, VIC Australia; 10 School of Population Health, University of New South Wales, Sydney, NSW Australia; 11 School of Psychology and Public Health, La Trobe University, Melbourne, Australia

**Keywords:** Injecting drug use, Prison, Opioids, Opioid agonist treatment, Harm reduction, Methadone, Buprenorphine, Primary care

## Abstract

**Background:**

A precipitous decline in health status among people recently released from prison is common. In Victoria, Australia, opioid agonist treatment (OAT) in the community involves frequent contact with primary care, potentially facilitating broader use of primary healthcare services. Among a cohort of men who injected drugs regularly pre-imprisonment, we estimated differences in rates of primary healthcare use and medication dispensation between people who did and did not receive OAT post-release.

**Methods:**

Data came from the Prison and Transition Health Cohort Study. Three-month post-release follow-up interviews were linked with primary care and medication dispensation records. Generalised linear models were fit with one exposure (OAT: none/partial/complete) for 13 outcomes relating to primary healthcare use, pathology testing, and medication dispensation, adjusted for other covariates. Coefficients were reported as adjusted incidence rate ratios (AIRR).

**Results:**

Analyses included 255 participants. Compared to no OAT use, both partial and complete OAT use were associated with increased rates of standard (AIRR: 3.02, 95%CI: 1.88–4.86; AIRR: 3.66, 95%CI: 2.57–5.23), extended (AIRR: 2.56, 95%CI: 1.41–4.67; AIRR: 2.55, 95%CI: 1.60–4.07) and mental health-related (AIRR: 2.71, 95%CI: 1.42–5.20; AIRR: 2.27, 95%CI: 1.33–3.87) general practitioner (GP) consultations, total medication (AIRR: 1.88, 95%CI: 1.19–2.98; AIRR: 2.40, 95%CI: 1.71–3.37), benzodiazepine (AIRR: 4.99, 95%CI: 2.81–8.85; AIRR: 8.30, 95%CI: 5.28–13.04) and gabapentinoid (AIRR: 6.78, 95%CI: 3.34–13.77; AIRR: 4.34, 95%CI: 2.37–7.94) dispensations, respectively. Partial OAT use was also associated with increased after-hours GP consultations (AIRR: 4.61, 95%CI: 2.24–9.48) and complete OAT use? with increased pathology utilisation (e.g. haematological, chemical, microbiological or immunological tissue/sample testing; AIRR: 2.30, 95%CI: 1.52–3.48).

**Conclusion:**

We observed higher rates of primary healthcare use and medication dispensation among people who reported partial and complete OAT use post-release. Findings suggest that access to OAT post-release may have a collateral benefit in supporting broader health service utilisation, underscoring the importance of retention in OAT after release from prison.

## Background

People in prison experience poorer physical and mental health than people in the general population [1, 2]. Conditions such as cardiovascular disease, blood borne viruses, substance use disorders and mental illness are all more prevalent among people in prison than the general population [1, 3–7]. Difficulties with obtaining secure housing [5, 8], re-establishing social support networks [9], substance use [10–12] and unemployment [13–15] are common following community re-entry. These challenges often intersect and compound disadvantage [16, 17], contributing to declines in physical and mental health [1, 18] and increased risk of mortality [19–21].

Early and regular contact with primary care has the potential to support improved physical and mental health among people recently released from prison [22–25]. For people experiencing opioid dependence, one mechanism likely to promote early and regular primary healthcare contact after release from prison is use of primary healthcare-based opioid agonist treatment (OAT). In some Australian jurisdictions, programmes that include short-term subsidisation of OAT dispensing fees, to support continuity of prison-based OAT in the community, require people continuing OAT post-release to present to a primary healthcare-based general practitioner (GP) within one week of release [26]. Community prescribing guidelines then encourage at least monthly OAT reviews with their GP [27], thereby affording regular opportunities to identify and respond to other concurrent health issues.

International studies of cohorts of people receiving OAT have found that retention in OAT is associated with increased rates of primary healthcare contact [28–31], use of pathology services [30], and receipt of prescription medication [29, 30, 32, 33]. Further, receipt of buprenorphine-based OAT prescribed in primary healthcare settings has been found to be associated with higher rates of chronic disease screening than in people prescribed OAT by psychiatric specialists [34], and a global systematic review and meta-analysis found that recent use of OAT was associated with elevated odds of recent hepatitis C testing and treatment uptake [35]. However, other studies have found that compared to the general population, people receiving OAT were less likely to receive chronic disease and cancer screening and imaging, despite attending primary healthcare services at greater rates [28, 31]. Few Australian studies have explored the relationship between OAT and broader primary healthcare engagement, reporting conflicting results. A study of COVID-19 vaccine uptake among people who injected drugs found increased odds of vaccine uptake among people receiving OAT [36]. Another reported that people who accessed community OAT programmes used GP and pathology services at approximately three times the rate of the general population, but accessed other health services and procedures (e.g. therapeutic procedures) at reduced rates [37]. In contrast, others found no association between current OAT use and past-month non-OAT GP contact [38].

How accessing OAT after release from prison influences the use of primary healthcare by people released from prison is unknown. Previous Australian research examining use of primary healthcare after release from prison [e.g. 25, 39, 40]. has not considered its relationship with use of OAT. Similarly, Australian studies of use of OAT after release from prison [e.g. 41–43] have not examined primary healthcare use. In the United States, Howell et al. [44] compared individual rates of healthcare use before a period of imprisonment during which participants enrolled in a state-wide prison-based OAT programme, with rates of healthcare use after release. They found that rates of non-acute outpatient care, hepatitis C antiviral, and OAT medication dispensations were significantly greater following imprisonment and OAT enrolment than pre-imprisonment, but no differences in rates of dispensation of psychiatric, human immunodeficiency antivirals or chemotherapy medications, and reduced dispensation of non-OAT opioid and benzodiazepine medication [44]. However, this study did not explore use of OAT post-release as an exposure.

To address these knowledge gaps, we used data from a prospective cohort study of men who injected drugs regularly prior to a period of imprisonment in Victoria, Australia, to compare rates of (1) standard community primary healthcare; (2) a broader range of community primary healthcare services; and (3) medication dispensation, between those who did and did not use OAT post-release.

## Methods

### Setting

This research was conducted in Victoria, Australia. Victoria had a population of 6,649,200 people at 30 June 2021 [45], making it Australia’s second most populous state. Victoria’s adult imprisonment rate rose from 110 people per 100,000 adult population in 2011 to 139 people per 100,000 adult population in 2021 [46]. OAT is available in all Victorian prisons. People receiving community-based OAT when imprisoned can continue OAT during imprisonment, and others may commence OAT in prison following clinical assessment [26]. Methadone and sublingual buprenorphine-naloxone were available as OAT medicines in Victorian prisons during data collection, but methadone is the Department of Justice and Community Safety (DJCS) preferred medicine for people commencing OAT in prison due a lower potential for diversion [26].

Shortly before release from prison, people receiving OAT are referred to a private community-based pharmacist with a prescription for daily OAT dispensation for a maximum of seven days, and also to a GP based in comprehensive primary healthcare for ongoing OAT prescribing. Victoria does not operate any public OAT clinics (prescribing or dispensing) [47]. GP visits are fully subsidised under the Medicare Benefits Schedule (MBS), Australia’s universal primary healthcare financing scheme, although some GPs may charge gap fees to the consumer. Consumers are required to attend a private pharmacy for supervised daily dosing. Consumers may be approved for unsupervised (i.e. take-home) doses by their prescribing GP following clinical assessment [27]. Consumers are charged a daily OAT dispensing fee by pharmacies, typically AUD$5/day [27]. In Victoria the DJCS subsidises the first 28 days of post-release dispensing fees [26]. Victorian community-based OAT treatment guidelines recommend intensive (minimum monthly) monitoring and review of OAT for people recently released from prison [27]. Urine screening for detection of illicit drug use is not a routine part of Victorian OAT programmes, but is one of a number of strategies available to prescribers when assessing suitability for unsupervised dosing [27].

### Participants and data sources

Data are from the Prison and Transition Health (PATH) Cohort Study, a prospective cohort study of 400 men recruited during a prison sentence and pending release in Victoria, Australia. Participants were recruited between September 2014 and May 2016 from one minimum-, one medium- and one maximum-security prison. PATH eligibility requirements included being sentenced (not pre-trial detention), aged 18 years or older, and self-reported at least monthly injecting drug use (IDU) in the 6 months preceding index imprisonment. Participants completed a baseline interview a median of 39 (IQR, 15–69) days prior to release. Follow-up interviews were conducted approximately three, 12 and 24 months after release from their index sentence. Detailed PATH methodology and cohort characteristics are published elsewhere [14, 48, 49].

In addition to interview participation, participants were asked if they wanted to consent to linkage of survey data to a range of health and social services databases. This analysis includes linked administrative data from the Medicare Benefits Schedule (MBS; Australia’s publicly-funded universal health financing scheme), Pharmaceutical Benefits Scheme (PBS; Australia’s publicly-funded universal medication financing scheme), National Death Index (NDI), and adult reimprisonment data from DJCS. Data linkage was conducted by the Australian Department of Human Services (MBS and PBS), DJCS (reimprisonment data), and the Australian Institute of Health and Welfare (NDI). Data custodians were supplied names (including known aliases), sex, dates of birth, last known addresses, corrections reference numbers (DJCS) and Medicare numbers, and used deterministic and probabilistic methods to link participant survey data with administrative data records [50]. Ethics approvals for PATH were obtained from the Alfred Hospital Ethics Committee (79/12) DJCS (CF/14/10169) and AIHW Ethics Committee (EO2014/1/77).

### Outcomes

To understand the breadth of health services and medications used by the study population, this study had 13 outcomes: five health service utilisation outcomes, and eight prescription medication outcomes.

MBS data compile records of a federal-government subsidised health service used by a person, with use of multiple health services per person per day possible. Each type of MBS-listed health service is assigned an item code, used by medical practitioners when making claims to the Commonwealth Government for payment for services [51]. Health services outcomes were total counts of MBS-recorded health services used by participants on or between the dates of index release and three-month follow-up. Health services outcomes were grouped according to MBS item codes, and included: (1) standard GP consultations (less than 20 min), (2) extended GP consultations (at least 20 min), (3) mental health GP consultations, (4) after-hours GP consultations (between 6 pm and 8am, on public holidays, and after 12 pm on a Saturday or on a Sunday), and (5) any pathology services. Specific MBS item codes for each outcome are provided in Appendix 1.

PBS data contain dispensing records of over 900 subsidised prescription medications; with data including all Section 85-listed (dispensed via community pharmacies) medications and some Section 100 (highly specialised medications dispensed via special arrangements) medications [52, 53]. Each record represents one medication dispensation event, such that dispensation of seven day’s medication, one month’s medication, or two month’s medication in one event all represent one PBS record. PBS data do not include the condition for which the medication was dispensed, therefore, data were grouped into eight medications-type outcomes according to first-level headings of the Anatomical Therapeutic Chemical (ATC) classification system [54], which groups medications according to pharmacological action and body system impacted. Medication outcomes, which were aggregate counts of PBS recorded medications dispensed to participants, included: (6) total dispensations, (7) nervous system, (8) anti-infective, (9) alimentary and metabolic system, and (10) other (comprising of all remaining ATC body systems) medications dispensed. Additionally, given the elevated risk of drug-related mortality among people recently released from prison [20, 21, 55–57], dispensation of the following specific nervous system medications were also examined: (11) opioids (non-OAT), (12) benzodiazepines, and (13) gabapentinoids.

### Primary exposure

**Fig. 1:**
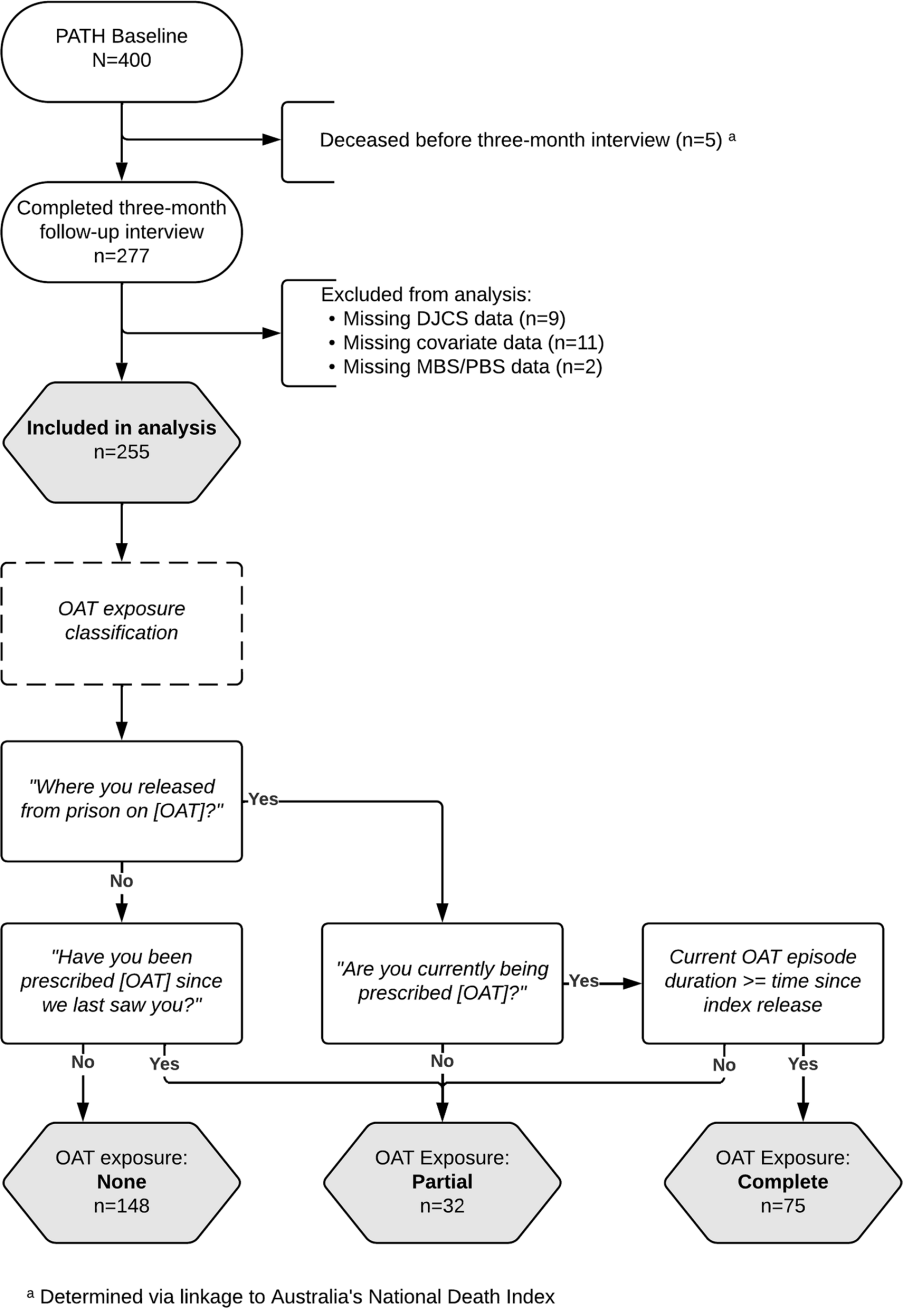
PATH participant inclusion and OAT exposure classification

The primary exposure for this study was the use of OAT after release from prison. As individual-level OAT dispensing data in Victoria were not collected in any administrative dataset during PATH observation, use of OAT was ascertained by self-report at the 3-month follow-up survey. At three-month follow-up, participants were asked: “*Were you released from prison on [OAT]?*”, *“Have you been prescribed [OAT] since we last saw you?”, “Are you currently being prescribed [OAT]?”,* and if currently on OAT at three-month interview *“How long [days/weeks/months] have you been on [OAT] for?”*. These answers were used to classify participants into one of three OAT use strata (none/partial/complete) as per Fig. 1 (below). Due to an insufficient number of participants prescribed sublingual buprenorphine-naloxone, analyses did not dissaggregate by OAT medicines.

### Covariates

Model covariates were selected following a review of literature relating healthcare use among people who use drugs [38, 58, 59], people recently released from prison [25], and access to healthcare in rural and regional Australia [60]. Covariates derived from baseline survey data included: age (years, continuous); Aboriginal and/or Torres Strait Islander (no/yes); ever diagnosed with a mental illness (no/yes); and ever diagnosed with a chronic health condition (no/yes). Covariates derived from three-month survey included: reporting fair or poor health status (poor or fair/good or excellent); reporting current accommodation as unstable (no/yes); times moved accommodation since index release (0/1 − 2/3 +); area of residence at 3-month interview (metropolitan/regional or rural/prison); main drug injected in the preceding 30 days (heroin/methamphetamine/heroin and methamphetamine/neither heroin nor methamphetamine); having a current support worker (no/yes); and current psychiatric distress assessed via the 12-Item General Health Questionnaire (GHQ-12; integer), scored according to the C-GHQ-12 scoring method [62]. Further covariate descriptions are available in Appendix 2.

### Data analysis

Among 400 PATH participants, five died prior to three-month follow-up (see Fig. 1). Among remaining participants, 277 (70%) completed a three-month follow-up interview. Of these, nine participants were excluded from analysis because DJCS linkage occurred between index release and three-month follow-up interview, resulting in incomplete reimprisonment data and preventing calculation of time in the community. Two participants who could not be linked with MBS or PBS data were also excluded. A complete case approach was used, resulting in the exclusion of a further 11 participants due to missing covariate data, resulting in a final analysis sample of 255 participants. Differences between excluded and included participants were examined for baseline sociodemographic variables using independent sample t-tests for continuous variables and chi-square tests for categorical variables.

Baseline descriptive statistics for sociodemographics were generated. Outcome data were skewed, so median and ranges are reported for each outcome. We considered the occurrence of zero outcomes in the data; we assumed that the probability of being able to access primary healthcare and prescription medication was greater than zero for all participants, such that all observed zeros were considered to occur by chance. Given these assumptions, and the over-dispersion of all outcomes, we used generalised linear models with a negative binomial distribution to estimate associations between the mean count of each outcome (that is, 13 models were estimated) and OAT use post-release, adjusted for other covariates. For interpretability we modelled the outcomes as rates, with days at risk in the community included in modelling as an exposure term (i.e. the count of each outcome during time at risk). As people are not eligible for MBS and PBS subsidies during imprisonment [65], time at risk (days) was calculated as time between release from index sentence and three-month follow-up interview, with any time spent reimprisoned deducted from this. Reimprisonment dates were determined via linkage to DJCS data; as DJCS data were unavailable beyond three-month follow-up interviews for the whole cohort to account for time in the community, all analyses examine the period between index release and three-month follow-up interview. Model estimates were reported as crude and adjusted incidence rate ratios (IRR and AIRR) with 95% confidence intervals (95%CI). Statistical significance was set at *p* < 0.05. All analyses were conducted using Stata 14.1 [66].

To assess whether relationships between use of OAT and primary healthcare use were impacted by the inclusion of participants who may not have used opioids in the observation period, and were therefore not indicated for OAT, we conducted additional sensitivity analyses. Each of the 13 models described above were refit with a restricted sample including only participants who reported at the 3-month follow-up survey any use of heroin or pharmaceutical opioids (e.g. methadone, buprenorphine, oxycodone; inclusive of prescribed and illicit/diverted) since last interview.

## Results

### Participant characteristics

The mean age of participants (*N* = 255) was 36 years (standard deviation: 8 years, see Table 1) and 15% identified as Aboriginal and/or Torres Strait Islander peoples. Most (91%) were born in Australia, 13% reported being employed prior to index imprisonment, and 20% had completed secondary school. Most (84%) reported ever having been diagnosed with a mental illness and 64% reported ever having been diagnosed with a chronic health condition. At three-month follow-up, 50% of participants were residing in a metropolitan area, and most reported their main drug injected in the past 30 days as methamphetamine (28%), heroin (12%) or a combination of the two (21%). Over half (58%) of participants reported no use of OAT since index release, 13% reported partial OAT use, and 29% reported complete OAT use. No statistically significant differences in baseline characteristics were found between participants included and excluded (*n* = 143) from analysis (see Appendix 3).

**Table 1:** Participant characteristics at baseline and three-month follow-up interview (N = 255)

	n (%^a^)
Baseline	
Age (mean, [SD^b^])	36 (8)
Aboriginal and/or Torres Strait Islander	40 (16)
Chronic health condition	170 (67)
Mental health condition	213 (84)
Follow-up	
Area of residence	
Metropolitan	129 (51)
Regional	84 (33)
Prison	42 (16)
Times moved accommodation	
0	110 (43)
1–2	75 (29)
3 +	70 (27)
Current accommodation unstable	40 (16)
Main drug injected in past 30 days	
Heroin	32 (13)
Methamphetamine	72 (28)
Methamphetamine and heroin	53 (21)
Neither methamphetamine or heroin	98 (38)
GHQ-12 score (median, [IQR^c^])	3 (1–6)
Self-reported fair or poor health	75 (29)
Current support worker	72 (28)
OAT exposure	
None	148 (58)
Partial	32 (13)
Complete	75 (29)

^a^Percentages may not add to 100% due to rounding; ^b^Standard deviation; ^c^Interquartile range

### Primary healthcare utilisation and OAT

Aggregate counts, summary statistics, and crude and adjusted IRR for each health service outcome (outcomes 1–5) stratified by post-release OAT use are shown in Table 2, with extended summary statistics available in Appendix 4. A total of 1113 GP consultations was observed, with 201 participants accessing at least one consultation. Standard GP consultations (*n* = 697, 63%) were the most commonly accessed, followed by extended GP consultations (*n* = 194, 17%).

**Table 2:** Item totals, median and range, for each level of opioid agonist treatment (OAT) use (None: *n* = 148, Partial: *n* = 32, Complete: *n* = 75), unadjusted (IRR) and adjusted incidence rate ratios (AIRR) comparing use of primary healthcare services across different levels of post-release OAT use

Outcome	Total count^a^	Median (range)	IRR (95%CI)	*p*-value	AIRR^b^ (95%CI)	*p*-value
1. Standard consultations	697	2 (0–30)				
OAT: None	227	1 (0–15)	REF		REF	
OAT: Partial	127	3.5 (0–19)	2.71 (1.73–4.26)	< 0.001	3.02 (1.88–4.86)	< 0.001
OAT: Complete	343	4 (0–30)	3.33 (2.39–4.66)	< 0.001	3.66 (2.57–5.23)	< 0.001
2. Extended consultations	194	0 (0–10)				
OAT: None	82	0 (0–10)	REF		REF	
OAT: Partial	33	1 (0–4)	2.07 (1.17–3.66)	0.012	2.56 (1.41–4.67)	0.002
OAT: Complete	79	1 (0–8)	2.19 (1.43–3.36)	< 0.001	2.55 (1.60–4.07)	< 0.001
3. Mental health consultations	136	0 (0–7)				
OAT: None	57	0 (0–7)	REF		REF	
OAT: Partial	27	0 (0–7)	2.20 (1.19–4.06)	0.012	2.71 (1.42–5.20)	0.003
OAT: Complete	52	0 (0–6)	2.00 (1.24–3.23)	0.004	2.27 (1.33–3.87)	0.003
4. After-hours consultations	86	0 (0–9)				
OAT: None	29	0 (0–4)	REF		REF	
OAT: Partial	26	0 (0–6)	4.85 (2.49–9.47)	< 0.001	4.61 (2.24–9.48)	< 0.001
OAT: Complete	31	0 (0–9)	2.22 (1.23–4.01)	0.008	1.56 (0.78–3.10)	0.207
5. Pathology items	353	0 (0–26)				
OAT: None	153	0 (0–10)	REF		REF	
OAT: Partial	37	0 (0–7)	1.24 (0.72–2.12)	0.434	1.26 (0.71–2.23)	0.426
OAT: Complete	163	0 (0–26)	2.51 (1.74–3.62)	< 0.001	2.30 (1.52–3.48)	< 0.001

^a^Between date of index release and three-month follow-up; ^b^Adjusted for age at baseline, Aboriginal or Torres Strait Islander, self-reported health status, main drug injected in the preceding 30 days, GHQ-12 score, area of residence, ever diagnosed with a mental illness, ever diagnosed with a chronic health condition, times moved since last interview, accommodation stability and current support worker

In each multivariable analysis, complete retention in OAT, compared to no OAT, was associated with an increased rate of standard GP consultations (outcome 1), extended GP consultations (outcome 2), and mental health GP consultations (outcome 3) and pathology services (outcome 5). Partial OAT, compared to no OAT, was associated with an increased rate of standard GP consultations (outcome 1), extended GP consultations (outcome 2), mental health GP consultations (outcome 3) and after-hours GP consultations (see Table 2).

Complete adjusted models for each healthcare utilisation outcome are available as (Appendix 5).

### Medication dispensation and OAT

Aggregate counts, summary statistics, and crude and adjusted IRR for each prescription medication outcome (outcomes 6–13) stratified by post-release OAT use are shown in Table 3. A total of 1188 dispensations occurred during observation, with 159 participants being dispensed at least one medication. Nervous system medications accounted for 75% (*n* = 894) of dispensations. Benzodiazepines accounted for 39% (*n* = 353) of nervous system medication dispensations; of these, diazepam (*n* = 224, 63%) and oxazepam (*n* = 78, 22%) were the most common benzodiazepines dispensed.

**Table 3:** Item totals, median and range, median and range for each level of opioid agonist treatment (OAT) use (None: *n* = 148, Partial: *n* = 32, Complete: *n* = 75), unadjusted (IRR) and adjusted incidence rate ratios (AIRR) comparing primary healthcare-based medication dispensation across different levels of post-release OAT use

Outcome	Total count^a^	Median (range)	IRR (95%CI)	*p*-value	AIRR^b^ (95%CI)	*p*-value
6. Total dispensations	1188	1 (0–49)				
OAT: None	548	1 (0–36)	REF		REF	
OAT: Partial	155	2 (0–28)	1.35 (0.88–2.07)	0.176	1.88 (1.19–2.98)	0.007
OAT: Complete	485	3 (0–49)	1.83 (1.34–2.50)	< 0.001	2.40 (1.71–3.37)	< 0.001
7. ATC: Nervous system	894	1 (0–49)				
OAT: None	338	0 (0–29)	REF		REF	
OAT: Partial	135	1.5 (0–27)	1.90 (1.22–2.95)	0.004	2.59 (1.61–4.16)	< 0.001
OAT: Complete	421	3 (0–49)	2.61 (1.89–3.60)	< 0.001	3.54 (2.48–5.03)	< 0.001
8. ATC: Anti-infectives	103	0 (0–15)				
OAT: None	79	0 (0–15)	REF		REF	
OAT: Partial	9	0 (0–3)	0.57 (0.25–1.27)	0.168	0.78 (0.33–1.83)	0.568
OAT: Complete	15	0 (0–2)	0.41 (0.22–0.76)	0.005	0.51 (0.26–1.00)	0.05
9. ATC: Alimentary & metabolic^c^	64	0 (0–11)				
OAT: None	41	0 (0–11)	REF		–	
OAT: Partial	2	0 (0–1)	0.22 (0.05–0.99)	0.048	–	–
OAT: Complete	21	0 (0–4)	1.10 (0.60–2.01)	0.755	–	–
10. ATC: Other	127	0 (0–10)				
OAT: None	90	0 (0–10)	REF		REF	
OAT: Partial	9	0 (0–4)	0.45 (0.20–1.01)	0.052	0.70 (0.29–1.70)	0.431
OAT: Complete	28	0 (0–8)	0.61 (0.36–1.03)	0.062	0.72 (0.39–1.33)	0.289
11. Opioids	125	0 (0–17)				
OAT: None	80	0 (0–17)	REF		REF	
OAT: Partial	10	0 (0–4)	0.58 (0.27–1.26)	0.167	0.89 (0.36–2.21)	0.797
OAT: Complete	35	0 (0–10)	0.94 (0.57–1.54)	0.801	1.08 (0.57–2.06)	0.808
12. Benzodiazepines	353	0 (0–45)				
OAT: None	74	0 (0–25)	REF		REF	
OAT: Partial	68	0 (0–27)	4.29 (2.55–7.22)	< 0.001	4.99 (2.81–8.85)	< 0.001
OAT: Complete	211	0 (0–45)	6.39 (4.30–9.50)	< 0.001	8.30 (5.28–13.04)	< 0.001
13. Gabapentinoids^d^	114	0 (0–14)				
OAT: None	28	0 (0–9)	REF		REF	
OAT: Partial	33	0 (0–14)	6.76 (3.55–12.87)	< 0.001	6.78 (3.34–13.77)	< 0.001
OAT: Complete	53	0 (0–11)	3.87 (2.24–6.70)	< 0.001	4.34 (2.37–7.94)	< 0.001

^a^Between date of index release and three-month follow-up; ^b^Adjusted for age at baseline, Aboriginal or Torres Strait Islander, self-reported health status, main drug injected in the preceding 30 days, GHQ-12 score, area of residence, ever diagnosed with a mental illness, ever diagnosed with a chronic health condition, times moved since last interview, accommodation stability and current support worker; ^c^An adjusted model was not estimated for associations between OAT and Alimentary & Metabolic dispensations due to an insufficient count among people reporting partial OAT; ^d^Adjusted model does not include covariate of main drug injected in the preceding 30 days due to an insufficient cell count in one level of this covariate

In each multivariable analysis, complete retention in OAT was associated with increased rates of total medication, nervous system, benzodiazepine, and gabapentinoid dispensations (see Table 3). Partial OAT was also associated with an increased rate of total medication, nervous system, benzodiazepine, and gabapentinoid dispensations.

Complete adjusted models for each prescription medication outcome are available as (Appendix 6).

A total of 195 (76% of 255) participants reported at least one episode of opioid use between baseline and three-month follow-up interviews, and were included in sensitivity analyses. Associations between use of OAT and primary healthcare among participants reporting opioid use were consistent with those observed in the primary analysis (see Table 4).

**Table 4:** Sensitivity analysis of item totals, median and range, median and range for each level of OAT use (None: *n* = 88, Partial: *n* = 32, Complete: *n* = 75), unadjusted (IRR) and adjusted incidence rate ratios (AIRR) comparing primary healthcare-based medication dispensation across different levels of post-release OAT use among participants who reported any opioid use since baseline interview (*N* = 195)

Outcome	Aggregate count^a^	Median (range)	IRR (95%CI)	*p*-value	AIRR^b^ (95%CI)	*p*-value
1. Standard consultations	608	2 (0–30)				
OAT: None	138	1 (0–15)	REF			
OAT: Partial	127	3.5 (0–19)	2.76 (1.70–4.47)	< 0.001	2.85 (1.70–4.77)	< 0.001
OAT: Complete	343	4 (0–30)	3.39 (2.33–4.94)	< 0.001	3.52 (2.35–5.27)	< 0.001
2. Extended consultations	152	0 (0–10)				
OAT: None	40	0 (0–10)	REF			
OAT: Partial	33	1 (0–4)	2.59 (1.38–4.85)	0.003	2.96 (1.51–5.79)	0.002
OAT: Complete	79	1 (0–8)	2.75 (1.66–4.54)	< 0.001	2.85 (1.66–4.89)	< 0.001
3. Mental health consultations	103	0 (0–7)				
OAT: None	24	0 (0–5)	REF			
OAT: Partial	27	0 (0–7)	3.26 (1.62–6.58)	0.001	3.13 (1.51–6.49)	0.002
OAT: Complete	52	0 (0–6)	2.97 (1.66–5.34)	< 0.001	2.71 (1.45–5.08)	0.002
4. After-hours consultations	73	0 (0–9)				
OAT: None	16	0 (0–2)	REF			
OAT: Partial	26	0 (0–6)	5.26 (2.46–11.22)	< 0.001	4.88 (2.12–11.25)	< 0.001
OAT: Complete	31	0 (0–9)	2.41 (1.21–4.79)	0.012	1.76 (0.79–3.92)	0.168
5. Pathology items	275	0 (0–26)				
OAT: None	75	0 (0–10)	REF			
OAT: Partial	37	0 (0–7)	1.52 (0.85–2.72)	0.154	1.49 (0.79–2.82)	0.216
OAT: Complete	163	0 (0–26)	3.09 (2.02–4.71)	< 0.001	2.76 (1.67–4.58)	< 0.001
6. Total prescriptions	925	1 (0–49)				
OAT: None	285	0 (0–36)	REF			
OAT: Partial	155	2 (0–28)	1.58 (1.00–2.50)	0.050	2.00 (1.21–3.29)	0.007
OAT: Complete	485	3 (0–49)	2.15 (1.51–3.05)	< 0.001	2.65 (1.79–3.93)	< 0.001
7. ATC: nervous system	762	1 (0–49)				
OAT: None	206	0 (0–29)	REF			
OAT: Partial	135	1.5 (0–27)	1.89 (1.19–3.03)	0.007	2.60 (1.55–4.37)	< 0.001
OAT: Complete	421	3 (0–49)	2.60 (1.82–3.73)	< 0.001	3.61 (2.38–5.47)	< 0.001
8. ATC: anti-infectives	53	0 (0–4)				
OAT: None	29	0 (0–4)	REF			
OAT: Partial	9	0 (0–3)	0.93 (0.39–2.21)	0.864	0.92 (0.37–2.32)	0.866
OAT: Complete	15	0 (0–2)	0.66 (0.33–1.34)	0.252	0.75 (0.34–1.65)	0.474
9. ATC: Alimentary & metabolic^c^	41	0 (0–6)				
OAT: None	18	0 (0–6)	REF			
OAT: Partial	2	0 (0–1)	0.31 (0.07–1.43)	0.134	–	–
OAT: Complete	21	0 (0–4)	1.53 (0.75–3.11)	0.241	–	–
10. ATC: Other	69	0 (0–9)				
OAT: None	32	0 (0–9)	REF			
OAT: Partial	9	0 (0–4)	0.79 (0.33–1.86)	0.589	0.75 (0.27–2.03)	0.568
OAT: Complete	28	0 (0–8)	1.07 (0.58–1.95)	0.836	0.87 (0.43–1.78)	0.708
11. Opioids	118	0 (0–17)				
OAT: None	73	0 (0–17)	REF			
OAT: Partial	10	0 (0–4)	0.37 (0.17–0.83)	0.015	0.60 (0.23–1.55)	0.289
OAT: Complete	35	0 (0–10)	0.61 (0.36–1.02)	0.059	0.62 (0.31–1.28)	0.197
12. Benzodiazepines	334	0 (0–45)				
OAT: None	55	0 (0–25)	REF			
OAT: Partial	68	0 (0–27)	3.60 (2.06–6.26)	< 0.001	4.59 (2.42–8.70)	< 0.001
OAT: Complete	211	0 (0–45)	5.35 (3.44–8.32)	< 0.001	7.19 (4.23–12.20)	< 0.001
13. Gabapentinoids^d^	102	0 (0–14)				
OAT: None	16	0 (0–8)	REF			
OAT: Partial	33	0 (0–14)	7.05 (3.39–14.69)	< 0.001	7.13 (3.21–15.86)	< 0.001
OAT: Complete	53	0 (0–11)	4.04 (2.11–7.75)	< 0.001	4.67 (2.32–9.38)	< 0.001

^a^Between date of index release and three-month follow-up; ^b^Adjusted for age at baseline, Aboriginal or Torres Strait Islander, self-reported health status, main drug injected in the preceding 30 days, GHQ-12 score, area of residence, ever diagnosed with a mental illness, ever diagnosed with a chronic health condition, times moved since last interview, accommodation stability and current support worker; ^c^An adjusted model was not estimated for associations between OAT and Alimentary & Metabolic dispensations due to an insufficient count among people reporting partial OAT; ^d^Adjusted model does not include covariate of main drug injected in the preceding 30 days due to an insufficient cell count in one level of this covariate

## Discussion

Among a cohort of men recently released from prison who reported regular IDU prior to imprisonment, we found that use of OAT in the post-release observation period was associated with increased use of standard and extended GP consultations in primary healthcare settings compared to those reporting no OAT use, consistent with existing literature [28–30, 44]. These elevated rates of primary healthcare attendance among people using OAT provide opportunities to address concurrent health priorities and support improved health outcomes among people recently released from prison. We also observed increased use of mental health GP consultations among people reporting partial or complete post-release OAT use, although the proportion of participants accessing these services during follow-up (32%) was low given the very high prevalence of mental health problems in the cohort. While previous studies have found elevated use of pathology services among people using OAT [30, 37], this was only observed among people reporting complete OAT use in this study. We also found increased use of after-hours GP services among people reporting partial OAT use, but not complete OAT.

While overall rates of total medication dispensation were higher among both partial and complete OAT groups than in the no OAT group, only medication dispensations for the nervous system were elevated when dispensations were analysed according to ATC group. We also observed increased rates of benzodiazepine and gabapentinoid prescription among people who accessed OAT, compared to people who did not. These findings were unexpected, given prior evidence of reduced benzodiazepine prescription among people using OAT [44, 67] and Victorian community-based OAT prescribing guidelines repeatedly cautioning against concurrent prescription of central nervous system depressants due to the risk of multiple drug toxicity and drug-related mortality [27].

### Implications

Studies have demonstrated the importance of integrated health and substance use treatment services in meeting the health needs of people who inject drugs [68, 69]. In our study, most GP consultations in the first three months after release from prison were standard consultations of less than 20 min duration. While MBS data do not include details of healthcare provided during GP consultations, short consultation durations may result in GPs prioritising initiation of OAT and/or stabilisation of OAT dose, potentiating other health issues being un- or under-treated. Diagnostic overshadowing is a well-recognised concern for people with complex health needs, after release from prison [70]. Routine use of extended consultations for people recently released from prison using OAT could support holistic individual health plans which concurrently address other health issues, including mental health, blood borne virus and chronic disease screening and treatment. However, the use of extended consultations may require increased OAT prescribing capacity. At June 2020, approximately eleven per cent of Victoria’s GPs were accredited to prescribe OAT [47, 71]. Currently in Victoria, GPs are required to undertake specialist training to prescribe methadone-OAT or buprenorphine-OAT to six or more patients, consisting of a short online module and a day-long module [72]. OAT accreditation requirements pose a barrier to recruiting new OAT prescribers and may cultivate a perception that OAT prescription is difficult [73]. The abolition of training requirements for buprenorphine-OAT, which carries a reduced risk of overdose [74], and the integration of methadone-OAT training into standard GP training and ongoing professional development, may increase the number of GPs prescribing OAT [73].

Our results add to the evidence supporting the health benefits of retention in OAT after release from prison. However, long-term retention in OAT after release from prison is rare [41, 43, 75–78]; indicating the need for reforms to OAT provision. Financial barriers such as dispensing fees [79–81] and indirect costs including transport [80] reduce OAT retention. Subsidising OAT in line with other PBS-listed prescription medications in Australia would improve health equity and affordability [80, 82], while increased access to unsupervised doses [80] may reduce indirect costs. Alternative OAT medications such as injectable diamorphine [83–85] are safe, effective and may support retention among people not retained in methadone- or buprenorphine-OAT. Routine clinical assessment supported by machine learning tools may support early identification of people at risk of premature OAT discontinuation, enabling early intervention [86], and online tools may also support retention [87–89]. Use of such programmes to support improved retention should be explored.

Improvements to the prison-to-community OAT referral process may provide additional opportunities to improve health among people receiving OAT after release from prison. In Victoria, prison discharge summaries, which are also used to support prison-to-community OAT referrals, contain fields for listing other concurrent physical and mental health issues which could be used to help GPs target care and assist in informing additional health screens. A randomised trial in Queensland, Australia, showed that active referral and provision of health summaries during the transition from prison to community increased primary care and mental healthcare contacts for at least 6 months post-release [39]. Currently, completion rates of non-OAT health information in OAT referrals in Victoria are unknown. However, research from New South Wales, Australia, found that women on OAT were no more likely to have prison health information transferred to community-based healthcare than women who were not using OAT [90].

Australian [e.g. 42, 55, 91] and international research [20, 21] has consistently shown an elevated risk of drug overdose among people recently released from prison. Both benzodiazepines [92, 93] and gabapentinoids [94, 95] are associated with an increased risk of drug-related morbidity and mortality. Although the majority of benzodiazepines dispensed to our cohort were lower potency medications, concurrent prescription of central nervous system depressants such as these may increase the risk of overdose among people using OAT post-release. This risk may be most pronounced among people who initiate or discontinue OAT in the immediate post-release period, as the weeks pre- and post-OAT are also associated with an elevated risk of opioid-related mortality [42, 96]. While previous research has found that retention in OAT after release from prison reduces the risk of opioid-related mortality [42, 97], it is unclear whether the high rates of benzodiazepine and gabapentinoid use may erode this protective effect. Future research should examine the reasons for benzodiazepine and gabapentinoid prescription during community re-entry, including whether they are prescribed and dispensed by the same healthcare providers, and any subsequent impact on the risk of drug overdose.

### Limitations and strengths

Several limitations should be considered when interpreting these findings. First, as the sample included only men who were regularly injecting drugs prior to imprisonment, these results cannot be generalised to other groups such as women, young people, people released from pre-trial detention, or people who do not inject drugs. In particular, for women in prison, the increased burden of many physical health, mental health and substance-related conditions [1, 3, 4], reproductive health needs [1] and differing patterns of healthcare access compared to men [5] may impact associations between OAT use post-release and primary healthcare utilisation. Replication of our findings in other settings will be required. Second, the sample size limited the precision of model estimates, especially for less frequent outcomes. Third, 123 participants who did not complete a 3-month follow-up interview were excluded from analysis. No differences were observed between participants included and excluded; however we cannot exclude the potential of attrition bias influencing our findings. Fourth, the absence of administrative OAT data may have introduced measurement and recall bias. Fifth, the PATH study design prevented determination of opioid dependence. As such, the ‘none’ OAT exposure group likely includes people who were not experiencing opioid dependence, either because they had ceased or reduced use, or were primarily using drugs other than opioids, and therefore unsuitable for OAT. However, the results of sensitivity analyses which excluded people who did not report any opioid use since baseline interview were consistent with primary results. Additionally, the absence of administrative OAT data also prevented temporal analysis of OAT exposure relative to health services use for participants reporting partial OAT use, which may have offered additional insight into patterns of health service use. Sixth, these findings represent associations between use of OAT and primary healthcare in the immediate post-release period. Future research should examine whether and how these associations change over time. Seventh, MBS data do not contain details of health matters discussed or treatments provided within consultations, preventing more detailed analysis. Eighth, given the absence of daily prescribed dose and medication indication from PBS data, estimation of mean medication dispensation rates are a proxy for healthcare use and not medication consumption per se. We note that the mean number of total medication dispensations was 4.6 (standard deviation: 7.8; see Appendix 3) over an average of 3.7 months of observation per person. Therefore, it is reasonable to use these data as a proxy for healthcare use. Finally, this analysis focused primarily on personal factors associated with health service utilisation. Despite these limitations, a key strength of this analysis was the linkage of self-report and administrative data. Administrative data provided unbiased data on frequency of primary healthcare use and medication dispensation, while self-report data allowed inclusion of covariates relating to housing stability and injecting drug use which are typically unavailable to analyses using only administrative data.

## Conclusion

People recently released from prison commonly experience significant declines in their physical and mental health. We observed higher rates of standard GP consultation in primary healthcare, and some evidence of broader primary healthcare use, including mental health consultations, among men with histories of IDU using OAT after release from prison. Retention in post-release OAT provides clear opportunities to address the concurrent health needs of people recently released from prison. Increased use of extended GP consultations among people recently released from prison who use OAT, and initiatives which support retention in OAT post-release, has the potential to support improved physical and mental health outcomes.

## Data Availability

The datasets analysed during this study are not publically available due to restrictions regarding the sharing of data for people involved with the criminal justice system in Victoria, Australia, but may be available upon request subject relevant ethics approvals.

## Appendix 1

(See Table

**Table 5:** Primary healthcare service outcomes and corresponding MBS item codes [51]

Outcome	Item codes
Standard consultations	3, 4, 23, 24
Extended consultations	36, 37, 44, 47
Mental health consultations	2700, 2701, 2712, 2713, 2715, 2717, 2721, 2725
After-hours consultations	5000, 5020, 5023, 5028, 5040, 5043, 5060
Pathology services	All category 6 item codes

5).

## Appendix 2

(See Table

**Table 6:** Covariate names, type, unit of measurement and additional description notes

Covariate	Type	Unit of measurement	Notes
Age	Continuous	Years	
Aboriginal and/or Torres Strait Islander	Binary	Yes/no	
Ever diagnosed with a mental illness	Binary	Yes/no	Includes depressive, anxiety, personality, and psychotic disorders
Ever diagnosed with a chronic health condition			Participants were classified as having a chronic health condition if they answered ‘yes’ at baseline interviews to ever having been diagnosed with a metabolic (e.g. diabetes), neurological (e.g. acquired brain injury, epilepsy), musculoskeletal (e.g. back injury, osteoarthritis), circulatory (e.g. hypertension, heart disease), respiratory condition (e.g. asthma, emphysema). Further, participants were also asked about ‘other health conditions’ at baseline; these were manually reviewed, and chronic conditions (e.g. psoriasis, gastrointestinal, cancer) reported here were also classified as chronic health conditions Infectious diseases, dental and hearing or vision conditions were not classified as, at the time of analysis, these conditions were unlikely to include routine treatment or care from general practitioners working in primary healthcare services
Fair or poor health status	Binary	Poor or fair/good or excellent	
Current accommodation unstable	Binary	Yes/no	
Times moved accommodation since index release	Ordinal	0/1 − 2/3 +	
Area of residence at three-month interview	Categorical	Metropolitan/regional or rural/prison	Determined by matching self-reported residential postcode to the Australian Bureau of Statistics’ Statistical Area Codes [61]. Participants who were reimprisoned at three-month interview were classified as ‘prison’
Main drug injected in the preceding 30 days	Categorical	Heroin/methamphetamine/heroin and methamphetamine/neither heroin nor methamphetamine	Neither heroin nor methamphetamine is comprised of people who did not report any injecting drug use (n = 90) and people who reported injecting drugs other than heroin or methamphetamine (n = 6) in the 30 days preceding three-month follow-up interview
Current support worker	Binary	Yes/no	
12-Item General Health Questionnaire (GHQ-12)	Integer	C-GHQ-12 scoring method; range: 0–12	The GHQ-12 is a standardised 12-item questionnaire which screens for poor psychiatric well-being over the preceding four weeks; higher scores indicate poorer psychiatric well-being condition [63, 64]

6).

## Appendix 3

(See Table

**Table 7:** Summary statistics of participant sociodemographic characteristics, stratified by analysis inclusion

	Excluded *N* = 145 (%)	Included *N* = 255 *n* (%)	*p*-value
Age (mean, SD^a^)	36 (9)	36 (8)	0.496^b^
Aboriginal and/or Torres Strait Islander			
No	119 (82)	215 (84)	0.561^c^
Yes	26 (18)	40 (16)	
Born outside of Australia			
No	125 (86)	233 (91)	0.105^c^
Yes	20 (14)	22 (9)	
Unemployed before index sentence			
No	14 (10)	33 (13)	0.338^c^
Yes	130 (90)	222 (87)	
Completed secondary school			
No	126 (87)	204 (80)	0.081^c^
Yes	19 (13)	51 (20)	
OAT^d^ at baseline interview			
No	81 (56)	154 (60)	0.376^c^
Yes	64 (44)	101 (40)	
Lifetime health condition			
No	49 (34)	85 (33)	0.925^c^
Yes	96 (66)	170 (67)	
Lifetime mental illness (N = 397)			
No	31 (22)	42 (16)	0.186^c^
Yes	111 (78)	213 (84)	
Main drug injected in the month prior to index imprisonment			
Heroin only	21 (14)	37 (15)	0.188^c^
Methamphetamine only	48 (33)	94 (37)	
Heroin and methamphetamine	63 (43)	87 (34)	
No heroin or methamphetamine	13 (9)	37 (15)	
GHQ-12^e^ score (mean, SD^a^)	5 (3)	5 (3)	0.435^b^

^a^Standard deviation, ^b^independent samples two-tailed t-test, ^c^chi-square test, ^d^opioid agonist treatment, ^e^General Health Questionnaire—12 Item

7).

## Appendix 4

(See Table

**Table 8:** Health services utilisation and medication dispensation item totals and extended summary statistics among PATH participants (N = 255)

Outcome	Total items	At least one contact or dispensation	Mean	Variance	SD^a^	Min	Q1^b^	Median	Q3^c^	Max
GP: Standard	697	172	2.73	13.47	3.67	0	0	2	4	30
GP: Extended	194	102	0.76	1.83	1.35	0	0	0	1	10
GP: Mental health	136	82	0.53	1.28	1.13	0	0	0	1	7
GP: After-hours	86	42	0.34	1.18	1.08	0	0	0	0	9
Pathology	353	62	1.38	10.07	3.17	0	0	0	0	26
Total scripts	1188	159	4.66	61.43	7.84	0	0	1	6	49
ATC: Nervous System	894	140	3.51	46.50	6.82	0	0	1	4	49
ATC: Anti-infectives	103	57	0.40	1.62	1.27	0	0	0	0	15
ATC: Alimentary & metabolic	64	26	0.25	1.01	1.00	0	0	0	0	11
ATC: Other	127	41	0.50	2.37	1.54	0	0	0	0	10
Opioids	125	39	0.49	3.11	1.76	0	0	0	0	17
Benzodiazepines	353	56	1.38	22.84	4.78	0	0	0	0	45
Gabapentinoids	114	28	0.45	3.00	1.73	0	0	0	0	14

^a^Standard deviation, ^b^25th percentile, ^c^75th percentile

8).

## Appendix 5

(See Table

**Table 9:** Participants (*N* = 255), total outcome counts (between date of index release and three-month follow-up), outcome counts stratified by model covariates and results of generalised linear models for health service outcomes, reported as adjusted incidence rate ratios (AIRR)

	Participants (*N* = 255)	1. Standard consultations (*n* = 697)		2. Extended consultations (*n* = 194)		3. Mental health consultations (*n* = 136)		4. After-hours consultations (*n* = 86)		5. Pathology items (*n* = 353)	
	*n* (%)	*n* (%)	AIRR (95%CI)	*n* (%)	AIRR (95%CI)	*n* (%)	AIRR (95%CI)	*n* (%)	AIRR (95%CI)	*n* (%)	AIRR (95%CI)
*Post-release OAT use*											
None	148 (58)	227 (33)	REF	82 (42)	REF	57 (42)	REF	29 (34)	REF	153 (43)	REF
Interrupted	32 (13)	127 (18)	3.02 (1.88–4.86)	33 (17)	2.56 (1.41–4.67)	27 (20)	2.71 (1.42–5.20)	26 (30)	4.61 (2.24–9.48)	37 (10)	1.26 (0.71–2.23)
Complete	75 (29)	343 (49)	3.66 (2.57–5.23)	79 (41)	2.55 (1.60–4.07)	52 (38)	2.27 (1.33–3.87)	31 (36)	1.56 (0.78–3.10)	163 (46)	2.30 (1.52–3.48)
Age (mean, [SD^a^])	36 (8)	–	1.03 (1.01–1.05)	–	1.04 (1.01–1.07)	–	1.03 (1.00–1.06)	–	1.00 (0.97–1.04)	–	1.06 (1.03–1.08)
Aboriginal and/or Torres Strait Islander	40 (16)	70 (10)	0.70 (0.43–1.13)	26 (13)	0.80 (0.44–1.47)	12 (9)	0.67 (0.32–1.42)	7 (8)	0.78 (0.30–2.03)	41 (12)	0.73 (0.42–1.25)
Self-reported fair or poor health	75 (29)	223 (32)	1.59 (1.07–2.36)	72 (37)	1.30 (0.80–2.13)	39 (29)	0.76 (0.43–1.34)	26 (30)	1.05 (0.51–2.15)	133 (38)	1.26 (0.82–1.94)
GHQ-12 Score	3 (1–6)	–	0.98 (0.93–1.03)	–	0.99 (0.92–1.06)	–	1.04 (0.96–1.13)	–	1.07 (0.97–1.17)	–	0.98 (0.92–1.05)
*Area of residence*											
Metro	129 (51)	394 (57)	REF	103 (53)	REF	82 (60)	REF	57 (66)	REF	205 (58)	REF
Regional	84 (33)	195 (28)	0.78 (0.54–1.15)	68 (35)	0.98 (0.60–1.58)	34 (25)	0.58 (0.34–1.01)	11 (13)	0.31 (0.14–0.68)	120 (34)	0.89 (0.58–1.36)
Prison/other	42 (16)	108 (15)	1.07 (0.64–1.79)	23 (12)	0.80 (0.40–1.60)	20 (15)	0.69 (0.32–1.47)	18 (21)	0.60 (0.24–1.52)	28 (8)	0.60 (0.32–1.13)
*Main drug injected in the last 30 days*											
Heroin	32 (13)	103 (15)	REF	31 (16)	REF	20 (15)	REF	12 (14)	REF	55 (16)	REF
Methamphetamine	72 (28)	139 (20)	1.14 (0.65–1.99)	44 (23)	1.09 (0.52–2.26)	33 (24)	1.23 (0.55–2.73)	12 (14)	0.58 (0.20–1.66)	51 (14)	0.99 (0.51–1.91)
Methamphetamine and Heroin	53 (21)	151 (22)	1.38 (0.79–2.40)	31 (16)	0.82 (0.40–1.71)	17 (13)	0.72 (0.31–1.66)	10 (12)	0.41 (0.14–1.21)	73 (21)	1.67 (0.90–3.11)
No heroin or methamphetamine	98 (38)	304 (44)	1.69 (1.00–2.87)	88 (45)	1.74 (0.88–3.42)	66 (49)	2.05 (0.99–4.27)	52 (60)	1.95 (0.80–4.78)	174 (49)	2.53 (1.40–4.56)
Self-reported lifetime chronic health condition	170 (67)	458 (66)	0.81 (0.58–1.15)	144 (74)	1.17 (0.73–1.88)	95 (70)	1.11 (0.66–1.88)	66 (77)	1.47 (0.74–2.93)	249 (71)	0.93 (0.62–1.38)
Self-reported lifetime mental health condition	213 (84)	585 (84)	1.15 (0.74–1.79)	177 (91)	2.04 (1.07–3.90)	121 (89)	1.64 (0.83–3.27)	75 (87)	1.18 (0.52–2.65)	308 (87)	1.51 (0.90–2.56)
Times moved since last interview (median, [IQR^b^])	1 (0–3)	–	0.91 (0.72–1.15)	–	1.11 (0.83–1.50)	–	0.94 (0.67–1.33)	–	1.21 (0.79–1.86)	–	1.07 (0.82–1.39)
Self-reported unstable accommodation	40 (16)	108 (15)	0.84 (0.49–1.44)	40 (21)	0.94 (0.47–1.88)	21 (15)	0.83 (0.37–1.89)	24 (28)	1.24 (0.50–3.06)	53 (15)	0.80 (0.43–1.47)
Current support worker	72 (28)	261 (37)	1.78 (1.25–2.52)	75 (39)	1.35 (0.85–2.13)	56 (41)	1.82 (1.11–3.00)	27 (31)	0.98 (0.52–1.85)	104 (29)	1.01 (0.67–1.52)
Model constant	–	–	0.00 (0.00–0.01)	–	0.00 (0.00–0.00)	–	0.00 (0.00–0.00)	–	0.00 (0.00–0.01)	–	0.00 (0.00–0.00)

^a^Standard deviation; ^b^Interquartile range

9).

## Appendix 6

(See Table

**Table 10:** Participants (*N* = 255), total outcome counts (between date of index release and three-month follow-up), outcome counts stratified by model covariates and results of generalised linear models for each prescription medication outcome, reported as adjusted incidence rate ratios (AIRR)

	Participants (*N* = 255)	6. Total Dispensation s(*n* = 1188)		7. ATC: Nervous system (*n* = 894)		8. ATC: Anti-infectives (*n* = 103)		9. ATC: Alimentary & Metabolic (*n* = 64)	
	*n* (%)	*n* (%)	AIRR (95%CI)	*n* (%)	AIRR (95%CI)	*n* (%)	AIRR (95%CI)	*n* (%)	AIRR (95%CI)
*Post-release OAT use*									
None	148 (58)	548 (46)	REF	338 (38)	REF	79 (77)	REF	41 (64)	–
Interrupted	32 (13)	155 (13)	1.88 (1.19–2.98)	135 (15)	2.59 (1.61–4.16)	9 (9)	0.78 (0.33–1.83)	np^a^	–
Complete	75 (29)	485 (41)	2.40 (1.71–3.37)	421 (47)	3.54 (2.48–5.03)	15 (15)	0.51 (0.26–1.00)	21 (33)	–
Age (mean, [SD^b^])	36 (8)	–	1.05 (1.03–1.07)	–	1.05 (1.03–1.07)	–	1.03 (1.00–1.06)	–	–
Aboriginal and/or Torres Strait Islander	40 (16)	129 (11)	0.53 (0.34–0.83)	92 (10)	0.54 (0.33–0.86)	10 (10)	0.56 (0.24–1.27)	12 (19)	–
Self-reported fair or poor health	75 (29)	431 (36)	1.61 (1.11–2.34)	310 (35)	1.59 (1.07–2.34)	53 (51)	1.91 (1.06–3.45)	20 (31)	–
GHQ-12 Score	3 (1–6)	–	0.97 (0.92–1.01)	–	0.99 (0.94–1.04)	–	0.96 (0.89–1.05)	–	–
*Area of residence*									
Metro	129 (51)	628 (53)	REF	478 (53)	REF	52 (50)	REF	36 (56)	–
Regional	84 (33)	401 (34)	0.99 (0.69–1.42)	281 (31)	0.94 (0.64–1.36)	38 (37)	1.14 (0.62–2.10)	23 (36)	–
Prison/other	42 (16)	159 (13)	1.09 (0.68–1.77)	135 (15)	1.17 (0.71–1.93)	13 (13)	1.42 (0.58–3.47)	5 (8)	–
*Main drug injected in the last 30 days*									
Heroin	32 (13)	137 (12)	REF	101 (11)	REF	5 (5)	REF	14 (22)	–
Methamphetamine	72 (28)	272 (23)	1.92 (1.12–3.29)	190 (21)	1.72 (0.98–3.03)	43 (42)	4.59 (1.47–14.28)	6 (9)	–
Methamphetamine and Heroin	53 (21)	201 (17)	1.66 (0.96–2.86)	160 (18)	1.66 (0.94–2.93)	14 (14)	2.37 (0.71–7.91)	10 (16)	–
No heroin or methamphetamine	98 (38)	578 (49)	2.62 (1.58–4.36)	443 (50)	2.62 (1.53–4.46)	41 (40)	3.92 (1.28–11.96)	34 (53)	–
Self-reported lifetime chronic health condition	170 (67)	899 (76)	1.19 (0.85–1.66)	646 (72)	0.91 (0.64–1.30)	84 (82)	1.93 (1.04–3.58)	58 (91)	–
Self-reported lifetime mental health condition	213 (84)	1053 (89)	1.80 (1.16–2.79)	789 (88)	2.00 (1.25–3.21)	90 (87)	1.05 (0.50–2.18)	56 (88)	–
Times moved since last interview (median, [IQR^c^])	1 (0–3)	–	0.85 (0.67–1.08)	–	0.85 (0.66–1.09)	–	0.95 (0.64–1.40)	–	–
Self-reported unstable accommodation	40 (16)	234 (20)	1.34 (0.79–2.29)	183 (20)	1.22 (0.70–2.12)	33 (32)	2.26 (0.98–5.20)	11 (17)	–
Current support worker	72 (28)	503 (42)	1.88 (1.35–2.62)	373 (42)	2.06 (1.47–2.91)	39 (38)	1.52 (0.86–2.68)	37 (58)	–
Model constant	–	–	0.00 (0.00–0.00)	–	0.00 (0.00–0.00)		0.00 (0.00–0.00)		–

	10. ATC: Other (*n* = 127)		11. Opioids (*n* = 125)		12. Benzodiazepines (*n* = 353)		13. Gabapentinoids (*n* = 114)	
	*n* (%)	AIRR (95%CI)	*n* (%)	AIRR (95%CI)	*n* (%)	AIRR (95%CI)	*n* (%)	AIRR (95%CI)
*Post-release OAT use*								
None	90 (71)	REF	80 (64)	REF	74 (21)	REF	28 (25)	REF
Interrupted	9 (7)	0.70 (0.29–1.70)	10 (8)	0.89 (0.36–2.21)	68 (19)	4.99 (2.81–8.85)	33 (29)	6.78 (3.34–13.77)
Complete	28 (22)	0.72 (0.39–1.33)	35 (28)	1.08 (0.57–2.06)	211 (60)	8.30 (5.28–13.04)	53 (46)	4.34 (2.37–7.94)
Age (mean, [SD^b^])	–	1.09 (1.06–1.13)	–	1.07 (1.03–1.10)	–	1.06 (1.03–1.09)	–	1.03 (0.99–1.07)
Aboriginal and/or Torres Strait Islander	15 (12)	0.76 (0.34–1.67)	8 (6)	0.38 (0.14–1.05)	36 (10)	1.02 (0.55–1.88)	14 (12)	0.76 (0.33–1.71)
Self-reported fair or poor health	48 (38)	1.18 (0.63–2.22)	66 (53)	3.11 (1.59–6.07)	95 (27)	1.55 (0.94–2.55)	43 (38)	1.41 (0.74–2.69)
GHQ-12 Score	–	0.92 (0.84–1.01)	-	0.98 (0.90–1.06)	–	0.95 (0.88–1.02)	–	0.99 (0.91–1.07)
*Area of residence*								
Metro	62 (49)	REF	81 (65)	REF	178 (50)	REF	71 (62)	REF
Regional	59 (46)	1.24 (0.69–2.22)	38 (30)	0.77 (0.42–1.44)	92 (26)	0.97 (0.58–1.62)	24 (21)	0.56 (0.28–1.10)
Prison/other	6 (5)	0.43 (0.15–1.20)	6 (5)	0.35 (0.11–1.10)	83 (24)	1.93 (1.02–3.66)	19 (17)	1.66 (0.75–3.67)
*Main drug injected in the last 30 days*								
Heroin	17 (13)	REF	27 (22)	REF	47 (13)	REF	np^a^	–
Methamphetamine	33 (26)	0.97 (0.39–2.39)	23 (18)	0.49 (0.21–1.17)	83 (24)	1.70 (0.81–3.59)	12 (11)	–
Methamphetamine and Heroin	17 (13)	0.81 (0.32–2.03)	17 (14)	0.50 (0.20–1.26)	62 (18)	1.28 (0.64–2.59)	16 (14)	–
No heroin or methamphetamine	60 (47)	1.31 (0.57–3.02)	58 (46)	1.05 (0.48–2.27)	161 (46)	1.53 (0.78–3.00)	85 (75)	–
Self-reported lifetime chronic health condition	111 (87)	2.51 (1.30–4.85)	101 (81)	1.88 (0.97–3.66)	225 (64)	0.77 (0.49–1.23)	96 (84)	1.85 (0.93–3.68)
Self-reported lifetime mental health condition	118 (93)	3.47 (1.40–8.60)	102 (82)	1.15 (0.54–2.48)	291 (82)	1.58 (0.87–2.88)	110 (96)	5.12 (1.66–15.73)
Times moved since last intervview (median, [IQR^c^])	–	0.88 (0.59–1.32)	–	0.95 (0.63–1.44)	–	1.10 (0.81–1.51)	–	0.66 (0.43–1.02)
Self-reported unstable accommodation	7 (6)	0.35 (0.12–1.02)	9 (7)	0.16 (0.05–0.50)	91 (26)	2.01 (1.00–4.04)	32 (28)	2.10 (0.88–5.02)
Current support worker	54 (43)	1.08 (0.61–1.91)	69 (55)	3.98 (2.34–6.76)	140 (40)	1.96 (1.25–3.08)	33 (29)	1.03 (0.56–1.91)
Model constant	–	0.00 (0.00–0.00)	–	0.00 (0.00–0.01)	–	0.00 (0.00–0.00)	–	0.00 (0.00–0.00)

^a^Not provided due to a small cell size (*n* < 5); ^b^Standard deviation; ^c^Interquartile range

10).
